# Adaptive laboratory evolution of *Salmonella enterica* in acid stress

**DOI:** 10.3389/fmicb.2023.1285421

**Published:** 2023-11-16

**Authors:** Mrinalini Ghoshal, Tyler D. Bechtel, John G. Gibbons, Lynne McLandsborough

**Affiliations:** ^1^Department of Microbiology, University of Massachusetts, Amherst, MA, United States; ^2^Department of Food Science, University of Massachusetts, Amherst, MA, United States

**Keywords:** *Salmonella enterica*, adaptive laboratory evolution (ALE), acetic acid stress adaptation, minimum inhibitory concentration (MIC), bacterial whole genome sequencing, missense mutations

## Abstract

**Introduction:**

Adaptive laboratory evolution (ALE) studies play a crucial role in understanding the adaptation and evolution of different bacterial species. In this study, we have investigated the adaptation and evolution of *Salmonella enterica* serovar Enteritidis to acetic acid using ALE.

**Materials and methods:**

Acetic acid concentrations below the minimum inhibitory concentration (sub-MIC) were used. Four evolutionary lineages (EL), namely, EL1, EL2, EL3, and EL4, of *S*. Enteritidis were developed, each demonstrating varying levels of resistance to acetic acid.

**Results:**

The acetic acid MIC of EL1 remained constant at 27 mM throughout 70 days, while the MIC of EL2, EL3, and EL4 increased throughout the 70 days. EL4 was adapted to the highest concentration of acetic acid (30 mM) and demonstrated the highest increase in its MIC against acetic acid throughout the study, reaching an MIC of 35 mM on day 70. The growth rates of the evolved lineages increased over time and were dependent on the concentration of acetic acid used during the evolutionary process. EL4 had the greatest increase in growth rate, reaching 0.33 (h^−1^) after 70 days in the presence of 30 mM acetic acid as compared to EL1, which had a growth rate of 0.2 (h^−1^) after 70 days with no exposure to acetic acid. Long-term exposure to acetic acid led to an increased MIC of human antibiotics such as ciprofloxacin and meropenem against the *S. enterica* evolutionary lineages. The MIC of ciprofloxacin for EL1 stayed constant at 0.016 throughout the 70 days while that of EL4 increased to 0.047. Bacterial whole genome sequencing revealed single-nucleotide polymorphisms in the ELs in various genes known to be involved in *S. enterica* virulence, pathogenesis, and stress response including *phoP, phoQ*, and *fhuA*. We also observed genome deletions in some of the ELs as compared to the wild-type *S*. Enteritidis which may have contributed to the bacterial acid adaptation.

**Discussion:**

This study highlights the potential for bacterial adaptation and evolution under environmental stress and underscores the importance of understanding the development of cross resistance to antibiotics in *S. enterica* populations. This study serves to enhance our understanding of the pathogenicity and survival strategies of *S. enterica* under acetic acid stress.

## 1. Introduction

Adaptive laboratory evolution (ALE) is a technique used to study the evolution of organisms in response to selective pressures over time. In laboratory settings, ALE is typically performed with organisms possessing a short generation time such as bacteria. Through ALE, bacteria are subjected to increasingly harsh conditions over several generations, allowing them to adapt and evolve in response to stress (Dragosits and Mattanovich, 2013). The technique of ALE has proven to be highly effective in providing a deeper understanding of the genetic mechanisms and processes involved in bacterial adaptation, as well as in facilitating research on the evolution of bacterial populations over time (Gresham and Dunham, 2014; LaCroix et al., 2017; Sandberg et al., 2019).

*Salmonella enterica* is a pathogenic bacterium that can cause serious foodborne illnesses in humans (Spector and Kenyon, 2012; Ren et al., 2015). It frequently encounters acidic conditions in the external environment and inside hosts, and has evolved strategies to survive and adapt to acid stress (Álvarez-Ordóñez et al., 2012). The use of organic acids for fermentation and flavoring in the food industry provides opportunities for contaminating *S. enterica* to become acid-adapted (Coban, 2020). Additionally, the use of acidified solutions for cleaning and sanitization in households and industries creates environments conducive to the development of acid adaptation and acid tolerance response in surviving *S. enterica* (Wang et al., 2019). In this study, we have used an evolutionary approach to understand the adaptation of *S. enterica* to acid stress by subjecting it to sub-inhibitory concentrations of acetic acid over more than 1,000 bacterial generations. Subsequently, we were able to analyze the bacterial genome and identify the genetic mutations resulting from growth under acidic conditions.

Adaptive laboratory evolution of *S. enterica* in environmental stresses is a relatively unexplored field, making our study pioneering and essential. The results of the study have implications for food safety since understanding the evolution of *Salmonella* populations with reduced acid susceptibility can impact the efficacy of food preservation methods that rely on acidification. Moreover, prior adaptation to acid stress has been reported to provide cross-protection against multiple other forms of stresses that are frequently encountered by the pathogen in industrial settings (Xu et al., 2008). In this study, we evaluate whether continued exposure to acetic acid provides cross-resistance in *S. enterica* populations against human antibiotics. Understanding the relationship between acetic acid exposure and antibiotic resistance is crucial in combating the global issue of antibiotic resistance. Analyzing the acid-adaptive capabilities of *S. enterica* will contribute to our knowledge of the dynamics of bacterial evolution in response to stress. Overall, this study emphasizes the importance of understanding the genetic basis of bacterial adaptation to acid stress which will help in the development of effective preventative strategies.

## 2. Materials and methods

### 2.1. Bacterial isolates and growth media

The bacterial isolate used in this study is *Salmonella enterica* subsp. enterica serovar Enteritidis (ATCC BAA-1045, phage type 30). This bacterial strain was originally isolated from a salmonellosis outbreak in raw almonds (Brandl et al., 2008). It has been reported to survive long-term desiccation and nutrient starvation stress, which makes it an interesting target to study its adaptation to acid stress (Deng et al., 2012). The bacterial strain was maintained as frozen stocks in a −80°C freezer in trypticase soy broth (TSB; Sigma-Aldrich) supplemented with 15% glycerol. Frozen cultures of *S. enterica* were revived by streaking on tryptic soy agar (TSA; Sigma-Aldrich) and incubated at 37°C for 18 to 20 h to create a bacterial lawn. The bacteria were further streaked on trypticase soy agar (TSA, Sigma-Aldrich). Single colonies of bacteria were then inoculated in tryptic soy broth (TSB, BD diagnostic systems) overnight for 18 h.

### 2.2. Quantification of MIC (minimum inhibitory concentration) of acetic acid

The MIC of acetic acid against the evolutionary lineages of *S*. Enteritidis was determined using the broth dilution method (Wiegand et al., 2008) with minor modifications. In brief, in 50 mL tubes, a bacterial inoculum of 10^7^–10^8^ CFU/ml was added in 20 mL TSB containing acetic acid concentrations from 24 to 34 mM. The acetic acid concentration in each tube was incremented by 1 mM. The tubes were incubated under shaking conditions at 37°C for ∼18 h. TSB with 200 mM acetic acid and TSB without any acetic acid were also inoculated with the bacteria and served as negative and positive controls, respectively. The OD_600_ was quantified after 20 h, and OD_600_ ≤ 0.1 was considered an inhibition of growth. To determine the MIC or minimum bactericidal concentration (MBC) of acetic acid, inoculum was taken from the tubes with OD_600_ ≤ 0.1 and plated on TSA plates. Recovery of growth was an indication that the acetic acid concentration was MIC. This experiment was repeated in triplicates for each of the replicates of all the evolutionary lineages. The minimum inhibitory concentration of acetic acid of the evolutionary lineages was determined every 5 days after the initiation of the ALE study.

### 2.3. Adaptive laboratory evolution of *S*. Enteritidis in acetic acid

At the beginning of the adaptive evolutionary study, *Salmonella enterica* subsp. enterica serovar Enteritidis (referred to as WT) was grown in TSB overnight. The overnight *S*. Enteritidis culture was used to quantify the MIC of acetic acid [explained in section 2.2. Quantification of MIC (minimum inhibitory concentration) of acetic acid]. The MIC of WT *S*. Enteritidis was quantified to be 27 mM. The WT culture was grown overnight and after 20 h, the bacterial optical density of the overnight culture was adjusted, and inoculum of 10^7^–10^8^CFU/mL was added to three 50 mL conical tubes containing 20 mL of TSB. These tubes were labeled as EL1a, EL1b, and EL1c. Here, EL stands for evolutionary lineage. Inoculum from the WT was also added to three tubes containing 26 mM acetic acid in TSB that were labeled EL2a, EL2b, and EL2c. EL1 served as our control evolutionary lineage, and EL2 served as our first evolutionary lineage that was grown and adapted to sub-MIC of acetic acid. The six tubes were incubated at 37°C for 18–20 h. Following that, the OD_600_ of the six tubes was measured and recorded. An inoculum of 10^6^ CFU/ml from each tube was collected and added to six new tubes for day 2. This process was repeated throughout the ALE study, and a similar inoculum was transferred to fresh tubes for each of the EL daily. The MIC of the six tubes was quantified every 5 days, and on day 20, the MIC of EL2 (all three replicates) increased to 29 mM. Hence, on day 20, inoculum from EL2a-EL2c was also added to three tubes containing 28 mM acetic acid (sub-MIC acetic acid) in TSB and labeled EL3a-EL3c. On day 30, the MIC of EL3 increased to 31 mM, and inoculum from EL3a-EL3c was added to three tubes containing 30 mM acetic acid in TSB and labeled EL4a-EL4c. The OD_600_ for each of the tubes was measured after growing them for 20 h at 37°C under shaking conditions. Samples from the previous day were stored at −80°C over the course of the evolutionary process. The number of bacterial generations ‘n’ was calculated using the following equation (Lee et al., 2011):

n=log(N/N)0/log(2).

where *N* is the final number of cells in the conical tube after 20 h at the time of passage to the next day’s tubes. *N*_0_ is the initial number of cells that are transferred to each conical tube at the beginning of ALE for that day. The initial and final numbers of cells were estimated daily by measuring the OD_600_ using a spectrophotometer and using plate counts. The generation number calculation assumes that each cell is viable, the death rate is negligible, the cells are growing exponentially throughout the ALE experiment, and the cells are dividing by binary fission.

### 2.4. Quantification of growth rate of the *S*. Enteritidis evolutionary lineages

The growth rate of the bacterial lineages was quantified using previously established methods (Herring et al., 2006; Hall et al., 2013) with some modifications. After 20 h of growth of the evolutionary lineages at 37°C, the inoculum was adjusted to approximately 10^4^CFU/ml and added inside the wells of a 96-well microtiter plate (Thermo Fisher Scientific, Waltham, MA, USA, model number 266120). Bacterial cultures collected at the end of day 2 and day 70 from the ELs were used to generate the growth curves inside the oCelloScope. For WT *S*. Enteritidis, EL1 and EL2, along with the bacterial inoculum, the wells also contained TSB with 26 mM acetic acid. For EL3 and EL4, the wells in the microtiter plate contained 28 mM and 30 mM acetic acid in TSB, respectively. The concentration of acetic acid added to the wells for each EL was 1 mM less than their MIC (sub-MIC acetic acid). The microtiter plates were placed inside the oCelloScope for 24 h to monitor the progression of bacterial growth. The exponential growth rate of all three replicates of each of the evolutionary lineages was measured. The background corrected absorption (BCA) algorithm and the BCA normalized algorithm of the software UniExplorer (version 10.1) were used to quantify the kinetics of bacterial growth. The kinetics of growth of the WT *S*. Enteritidis and all the evolutionary lineages in TSB without acetic acid stress were also monitored using the oCelloScope.

### 2.5. Quantification of MIC of antibiotics against the *S*. Enteritidis evolutionary lineages

The MIC of the following antibiotics: vancomycin, meropenem, ciprofloxacin, gentamycin, and streptomycin, were determined using MTS strips (MIC test strips, Liofilchem) (Matuschek et al., 2018; van den Bijllaardt et al., 2018). Antibiotics with different mechanisms of action were chosen to gain a thorough understanding of their effects on the evolutionary lineages. Vancomycin and meropenem inhibit bacterial cell wall biosynthesis (Yarlagadda et al., 2018), ciprofloxacin inhibits bacterial DNA synthesis, and gentamycin and streptomycin are known to inhibit bacterial protein synthesis (Athamneh et al., 2014).

The test strips for each antibiotic had a concentration range from 0.016 to 256 μg/ml. Overnight cultures of the evolutionary lineages were grown in their corresponding acetic acid concentrations, and the next day, the OD_600_ was adjusted to 10^7^CFU/mL in TSB. Cotton swab applicators were dipped in the bacterial cultures and streaked on TSA to create bacterial lawns. The MTS test strips were carefully positioned at the center of the TSA plates using sterile forceps. The plates were incubated for 18–20 h at 37°C, following which the zones of inhibition around the test strips were recorded. The minimum concentration of the antibiotic, which caused inhibition of bacterial growth, was denoted as the MIC. This experiment was repeated in triplicates for each of the replicates of all the evolutionary lineages. MTS test strips have been used successfully for determining the MIC of antibiotics against different pathogens, and the strips have been reported to provide MIC values similar to those obtained using broth microdilution methods (Hakvoort et al., 2020; Koeth et al., 2022).

### 2.6. Bacterial whole-genome sequencing

The WT strain of *S*. Enteritidis and evolved lineages after day 70 of the ALE study were streaked on TSA and incubated overnight at 37°C. Genomic DNA extraction and sequencing were performed at SeqCenter (Pittsburgh, PA, USA). For the evolved lineages, after 70 days, the liquid cultures were plated on TSA, and colonies were randomly chosen for sequencing. DNA extraction was performed using Zymo DNA Miniprep (bead-beating lysis). For the WT strain, sample libraries were generated using the Illumina DNA Prep kit and IDT 10bp UDI indices. Sequencing was performed on a NextSeq 2000 sequencer, producing 151-bp paired-end reads. Low-quality reads were trimmed, and adapter sequences were removed from Illumina sequences using bcl-convert version 3.9.3. Oxford Nanopore Technologies (ONT) PCR-free ligation library preparation was also used to generate long sequence reads. ONT reads were adapted and quality-trimmed using porechop33 version 0.2.3_seqan2.1.1 (RRID:SCR_016967). Unicycler version 0.4.8 was used to produce a hybrid assembly from the Illumina and ONT reads. Assembly quality was assessed using QUAST version 5.0.2 (Gurevich et al., 2013). The NCBI Nucleotide BLAST database was then used to characterize the complete genome assembly and identify plasmid sequences. Gene models were predicted with functional annotations using Prokka version 1.14.5 with default parameters + ‘–rfam’ (Tatusova et al., 2016; Haft et al., 2018). Culturing and DNA extraction of the evolved lineages were performed using the same procedure as the WT, but 151-bp paired-end libraries were generated using only Illumina sequencing as described above.

### 2.7. Genome assembly and identification of polymorphisms

All software mentioned below was used with default parameters unless specified otherwise. BWA version 0.7.15 (RRID:SCR_010910) was used to map the evolved lineages resequencing data against the ancestral reference genome (Li, 2013). Samtools (v 1.14) (RRID:SCR_002105) was used to index the sorted BAM files. Bamaddrg was used to add read groups to the indexed and sorted BAM files (Danecek et al., 2021). Joint genotyping of the evolved strains was then performed using Freebayes (v 1.3.1) (RRID:SCR_010761) (Garrison and Marth, 2012). GATK (v. 4.0.6) (RRID:SCR_001876) was used to convert the resulting VCF files into a table format (McKenna et al., 2010). A SnpEff database was built for the *S.* Enteritidis BAA-1045 WT reference genome, and SNP annotation prediction was performed in the evolved strains using SnpEff (v. 4.1) (Cingolani et al., 2012). A second variant calling software, Breseq (v. 0.35.4) (RRID:SCR_010810), was used by SeqCenter to align and compare evolved lineage sequence reads to the ancestor (Deatherage and Barrick, 2014). Assemblies were generated from the Illumina reads of each evolved lineage using SPAdes (v. 3.13.1) (RRID:SCR_000131) with the parameters, “-k 21,33,55 –careful” (Anton Bankevich et al., 2012). Large-Scale Blast Score Ratio (LS-BSR) was used to investigate gene presence/absence mutations using default parameters, along with additional parameters “-b blastn -c cdhit” (Sahl et al., 2014). Read-depth analysis was performed using Samtools (v. 1.14).

### 2.8. Statistical analyses

All bacterial growth measurements were biologically triplicated, and their differences were examined by a two-tailed *t*- test assuming unequal variance (Welch’s *t*- test). GraphPad Prism was used to generate all the graphs in this project. Statistical significance was calculated with GraphPad Prism using two-way ANOVA. A *p*-value of ≤ 0.05 was considered statistically significant.

## 3. Results

### 3.1. Experimental design of the ALE study

**FIGURE 1:**
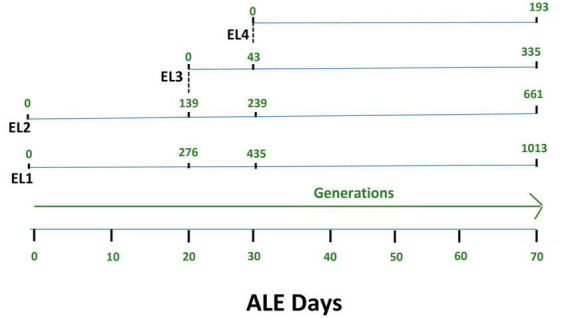
Adaptive evolution of *S*. Enteritidis evolutionary lineages (EL) with daily transfers in acetic acid. The MIC of acetic acid of wild-type *S*. Enteritidis was quantified to be 27 mM, and hence, EL2 was grown in 26 mM acetic acid (sub-MIC) while EL1 was grown in no acid stress (control group). The MIC of EL1 and EL2 was quantified every 5 days. On day 20, the MIC of EL2 increased to 29 mM. So, we started EL3 from EL2, and it was grown in 28 mM acetic acid with daily transfers for the remainder of the ALE study. On day 30, the MIC of EL3 went up to 31 mM; hence, we started EL4 from EL3, and EL4 was grown in 30 mM acetic acid with daily transfers until day 70 of the ALE study. The OD_600_ for each of the EL was quantified every day before and after daily transfers, and the values were used to quantify the number of generations. The number of generations of each of the ELs has been shown in green.

**FIGURE 2:**
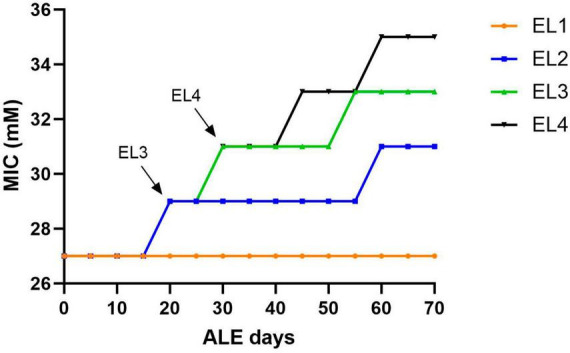
Change in acetic acid MIC of the ELs of *S*. Enteritidis. The MIC of acetic acid of the evolutionary lineages was measured every 5 days until day 70 of the ALE study. Bacterial cultures (1 mL) were collected from the three replicates of EL1–EL4 every 5 days to quantify the MIC of acetic acid. The arrows indicate the initiation of EL3 and EL4 from EL2 and EL3, respectively. The evolutionary lineage EL3 was initiated from EL2 (on ALE day 20) when the acetic acid MIC of EL2 increased for the first time and a similar process was used for the initiation of EL4 (on ALE day 30).

*Salmonella* Enteritidis was adapted in different concentrations of acetic acid in TSB over 70 days. Figure 1 outlines the evolution of four lines of *S. enterica* in acetic acid, which we are calling “evolutionary lineages” (EL). The MIC of acetic acid for wild-type (WT) *S.* Enteritidis was 27 mM at the beginning of the experiment (Figure 2). On day 0, two ELs were developed from WT *S.* Enteritidis. In the first EL, EL1, *S.* Enteritidis was grown in TSB without any acid stress, and in the second EL, EL2, *S.* Enteritidis was grown in 26 mM acetic acid (sub-MIC) in TSB. Each EL was grown in triplicates at 37°C for 18 h daily before they were transferred into fresh media as described in the section “2. Materials and methods”. The MIC of acetic acid of EL1 and EL2 was measured every 5 days, and the OD_600_ was monitored daily before transfer. After 20 ALE days, the MIC of acetic acid for EL2 rose to 29 mM, and a new lineage, EL3, was created from EL2 (Figure 1). An inoculum of 10^8^CFU/ml was transferred from the three replicates of EL2 to generate three replicates of EL3. EL3 was grown in 28 mM acetic acid for the remainder of the experiment. Here, ALE days refer to the number of days that have passed since the initiation of the ALE study. After 30 ALE days, the MIC of EL3 increased to 31 mM, and a new EL, EL4, was initiated (Figure 1). EL4 was grown in 30 mM acetic acid in TSB with daily transfers in fresh media. The number of generations for each EL was quantified from OD_600_ values and is shown in Figure 1. After 70 days, the adaptive evolution process was halted, and all the ELs were frozen at −80°C. The MIC of acetic acid of EL2–EL4 was found to increase over time throughout the duration of the ALE study (Figure 2). The MIC of acetic acid in EL1 remained constant throughout the study, and the highest increase in the MIC of acetic acid was observed in EL4.

### 3.2. Effect of ALE on the growth rate of the adapted evolutionary lineages

**FIGURE 3:**
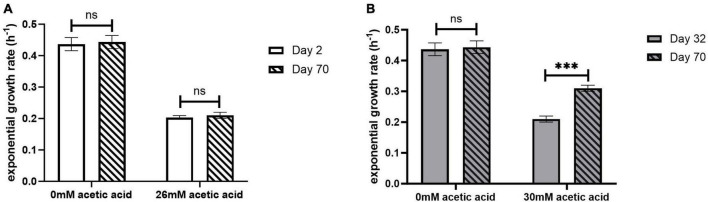
Exponential growth rate of EL1 **(A)** quantified on ALE days 2 and 70 and EL4 **(B)** quantified on ALE days 32 and 70 of *S*. Enteritidis evolutionary lines. ALE day 32 was the second day after the initiation of EL4 from EL3. *p*-value of 0.05 was considered to be statistically significant using two-tailed *t*-test. (***p < 0.001, ns, non-significant, = *p* > 0.05).

The exponential growth rate of the adapted ELs was evaluated in the presence and absence of acetic acid using the oCelloScope (Figure 3 and Table 1). The oCelloScope has previously been used to study changes in growth rates after treatment with antimicrobial compounds (Fredborg et al., 2013; Canali et al., 2018; Ghoshal et al., 2022b). The growth rates were quantified on ALE days 2 for EL1 and EL2 and for ALE days 22 and 32 for EL3 and EL4, respectively. ALE days 22 and 32 were 2 days after the initial transfer of EL3 and EL4, respectively. Growth rates of all ELs were calculated from the exponential growth phase of the growth curves. The growth curves of EL1 and EL4 generated using the oCelloScope are shown in Supplementary Figure 1.

**TABLE 1:** Growth rates of adapted evolutionary lines.

Acetic acid used during ALE study	Acetic acid used for growth rate determination	Evolutionary lineages	Growth rate without acetic acid (h^−1^)		Growth rate with acetic acid (h^−1^)	
			**Day 2**	**Day 70**	**Day 2**	**Day 70**
0 mM	26 mM	EL1a	0.40	0.40	0.20	0.22
		EL1b	0.44	0.41	0.21	0.21
		EL1c	0.42	0.41	0.19	0.20
			**Day 2**	**Day 70**	**Day 2**	Day 70**
26 mM	26 mM	EL2a	0.43	0.43	0.21	0.23
		EL2b	0.41	0.43	0.20	0.23
		EL2c	0.43	0.43	0.20	0.24
			**Day 22**	**Day 70**	**Day 22**	Day 70**
28 mM	28 mM	EL3a	0.45	0.42	0.21	0.26
		EL3b	0.42	0.42	0.22	0.28
		EL3c	0.44	0.43	0.20	0.28
			**Day 32**	**Day 70**	**Day 32**	Day 70***
30 mM	30 mM	EL4a	0.47	0.45	0.21	0.31
		EL4b	0.46	0.47	0.20	0.32
		EL4c	0.45	0.47	0.20	0.33

Growth rates of the evolutionary lineages were quantified using the oCelloScope. Growth rates have been compared between ALE days 2 and 70 for EL1 and EL2, ALE days 22 and 70 for EL3, and ALE days 32 and 70 for EL4 in the presence and absence of acetic acid. For EL3 (a–c) and EL4 (a–c), days 22 and 32 were 2 days after the start of the evolutionary lines, respectively. For quantification of statistical significance, growth rates on day 2 for EL1 and EL2, day 22 for EL3, and day 32 for EL4 have been used as controls. Growth rates of the controls have been compared to that on ALE day 70 for EL1–EL4 in the presence and absence of acetic acid. Although EL1 was not adapted to acetic acid during the evolutionary process, for determination of growth rate using the oCelloScope, EL1 (a–c) and WT were treated with 26 mM acetic acid. For WT, the growth rates with and without 26 mM acetic acid were 0.21 (h^−1^) and 0.40 (h^−1^), respectively. Growth rates of all three replicates of EL1, EL2, EL3, and EL4 on ALE day 70 in the absence of acetic acid were found to be statistically non-significant (*p* > 0.05) as compared to the controls. Growth rates of all three replicates of EL1 between days 2 and 70 in the presence of 26 mM acetic acid were also found to be statistically non-significant (*p* > 0.05). However, the growth rates of EL2, EL3, and EL4 on ALE day 70 in the presence of 26, 28, and 30 mM acetic acid, respectively, were found to be statistically significant as compared to their control growth rates. Asterisks (*) in the table indicate significant differences in growth rates in comparison with the controls (**p < 0.01 and ***p < 0.001).

No significant differences were observed in the growth rate after days 2 and 70 for EL1 (*p* > 0.05) (Figure 3A), while for EL4, the growth rate in 30 mM acetic acid on day 70 was significantly higher than that on day 32 (*p* < 0.001) (Figure 3B). However, no significant differences in growth rates in the absence of acetic acid were observed in either EL1 after days 2 and 70 or in EL4 after days 32 and 70 (*p* > 0.05). Additionally, the exponential growth rates of EL2 and EL3 have been represented graphically in Supplementary Figure 2. The exponential growth rate of all the ELs 2 days after their initiation and after 70 ALE days in the presence and absence of acetic acid is shown in Table 1. The results indicate that the growth rates on ALE day 70 were significantly higher than those on days 2, 22, and 32 for EL2 (*p* < 0.01), EL3 (*p* < 0.01), and EL4 (*p* < 0.001), respectively. On day 70, EL4 had the highest growth rate in 30 mM acetic acid followed by EL3 in 28 mM acetic acid and EL2 in 26 mM acetic acid. The lowest growth rate was observed for EL1 in the presence of 26 mM acetic acid after day 2, and no change in its growth rate was observed after day 70 (*p* > 0.05).

### 3.3. Effect of ALE on the MIC of human antibiotics in the evolutionary lineages

**FIGURE 4:**
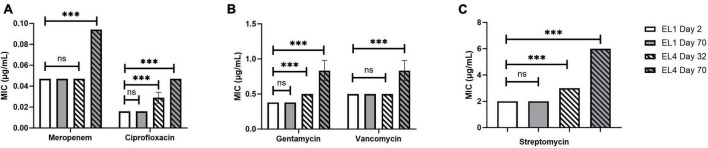
Change in MIC of human antibiotics against the *S*. Enteritidis evolutionary lineages EL1 (quantified on ALE days 2 and 70) and EL4 (quantified on ALE days 32 and 70). The antibiotics tested are meropenem and ciprofloxacin **(A)**, gentamycin and vancomycin **(B)**, and streptomycin **(C)**. EL1 was grown and adapted in TSB without stress throughout the ALE study, and EL4 was adapted in 30 mM acetic acid with initial transfer from EL3 on ALE day 30. *p*-value of 0.05 was considered to be statistically significant using two-tailed *t*-test. (***p < 0.001, ns, non-significant, = *p* > 0.05).

The MIC of ciprofloxacin, gentamycin, meropenem, streptomycin, and vancomycin was quantified in all the ELs using MTS (MIC test strips). MIC values of the antibiotics were quantified every 10 days starting from 2 days after the initiation of the ELs and ending after ALE day 70. The MIC of the antibiotics tested against EL1 and EL4 is shown in Figure 4. There were no significant differences in the MIC values of the antibiotics against EL1 between days 2 and 70. However, there was a significant difference in the MIC of ciprofloxacin (*p* < 0.05), gentamycin (*p* < 0.01), and meropenem (*p* < 0.001) against EL4 between days 32 and 70. Significant differences were also observed in EL3 against ciprofloxacin (*p* < 0.001) between ALE day 22 (2 days after the initiation of the evolutionary lineage) and day 70 of the ALE study. The MIC values of antibiotics against all the ELs are reported in Supplementary Table 1.

### 3.4. Comparative genomic analysis of acetic acid-evolved lineages

Genomic analysis of the evolutionary lineages was conducted after 70 ALE days to investigate the genetic determinants of the increased MIC against acetic acid and the human antibiotics tested. We generated a complete genome assembly of the WT *S.* Enteritidis using Illumina and Oxford Nanopore Technologies (ONT) sequencing. WT *S*. Enteritidis strain BAA-1045 whole-genome Illumina and Oxford Nanopore Technologies sequencing data are available for wild-type and evolved lineages (Illumina only) under the NCBI BioProject PRJNA1007881. The genome was assembled into four contigs consisting of the circular genome (∼4.67 Mb) and three plasmids with approximate sizes of 60 kb (*S.* Enteritidis strain RM2968 plasmid pRM2968-2), 56 kb (*S.* Enteritidis strain 56-3991 plasmid pSE56-3991), and 6.2 kb (*E. coli* strain RHB18-C20 plasmid pRHB18-C20_4) based on the best hits (*e*-value < 0.01, query coverage >99%, and percent identity >99.9%) against the NCBI Nucleotide Collection (nt) database (Johnson et al., 2008).

**FIGURE 5:**
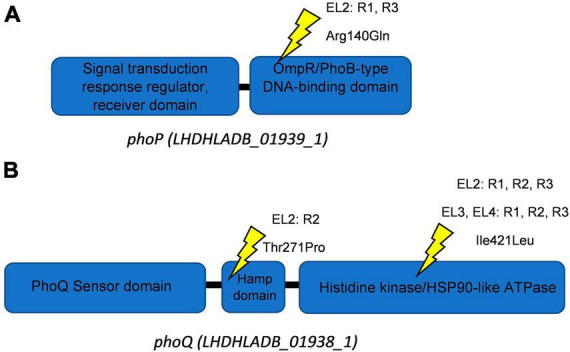
Schematics of PhoQ and PhoP proteins. Ellipses show protein domains with their respective Interpro identifiers for PhoP **(A)** and PhoQ **(B)**. Yellow symbols represent locations of missense mutations. The EL (evolutionary lineage), replicate (each EL had three replicates namely R1-R3), and amino acid substitution are provided for each site.

This high-quality genome assembly and annotation was used as a reference to map Illumina whole-genome sequencing data for each EL. The mutations observed within each evolved lineage are detailed in Table 2. We identified 8, 10, 3, and 5 base substitutions in EL1, EL2, EL3, and EL4, respectively. Base substitutions were observed in a total of 15 different genes (10 missense; 3 synonymous; 2 frameshift). Notably, missense mutations were observed in the histidine kinase gene, *phoQ*, among lineages EL2, EL3, and EL4. Moreover, missense mutations were observed in EL2 within *phoP*, which is the regulatory protein of *phoQ*. For the PhoP protein, mutations were observed in the DNA-binding domain, while in the PhoQ protein, mutations occurred in the HAMP domain and the histidine kinase domain (Figure 5).

**TABLE 2:** Mutations in evolutionary lines after adaptation in acetic acid for 70 ALE days identified using genomic analysis.

Position	Gene	Mutation	Ancestor allele	Evolved allele	Protein product	EL1	EL2	EL3	EL4
869465	*ptsP*	Missense	T	C	Phosphoenolpyruvate-dependent phosphotransferase system	R3: p.Tyr290His	R2: p.Tyr290His	X	x
2024345	*nimT*	Synonymous	C	T	2-nitroimidazole transporter	R1: p.Leu151Leu R2: p.Leu151Leu R3: p.Leu151Leu	R2: p.Leu151Leu	x	x
923432	*barA*	Missense	T	G	Signal transduction histidine-protein kinase BarA	x	R2: p.Thr268Pro	x	x
1061536	*alaS*	Missense	C	T	Alanine—tRNA ligase	x	R2: p.Gly272Gly	x	x
1973318	*phoQ*	Missense	T	G	Virulence sensor histidine kinase PhoQ	x	R2: p.Thr271Pro	x	x
1974384	*phoP*	Missense	C	T	Virulence transcriptional regulatory protein PhoP	x	R1: p.Arg140Gln R3: p.Arg140Gln	x	x
2015140	*zinT*	Frameshift	TAT	TAAT	Metal-binding protein ZinT	x	R2: p.Tyr200fs	x	x
3402681	*ybaY*	Frameshift	GTTTCA	GTTCA	putative lipoprotein YbaY	x	R1: p.Lys135fs	x	x
3675976	*fhuA*	Missense	G	T	Ferrichrome outer membrane transporter/phage receptor	x	R1: p.Ser318Arg R3: p.Ser318Arg	x	x
4348457	*rpoB*	Missense	A	C	DNA-directed RNA polymerase subunit beta	x	R1: p.Ser662Ala R3: p.Ser662Ala	x	x
1745485	*rfbE*	Missense	A	T	CDP-paratose 2-epimerase	x	x	R1: p.Asn220Tyr R2: p.Asn220Tyr R3: p.Asn220Tyr	R1: p.Asn220Tyr R2: p.Asn220Tyr R3: p.Asn220Tyr
1972868	*phoQ*	Missense	T	G	Virulence sensor histidine kinase PhoQ	x	x	R1: p.Ile421Leu R2: p.Ile421Leu R3: p.Ile421Leu	R1: p.Ile421Leu R2: p.Ile421Leu R3: p.Ile421Leu
3776974	*thiB*	Missense	AACGGTG ACGGTGA	AACGGTGACG GTGACGGTGA	Thiamine-binding periplasmic protein	x	x	R1: p.Val193_Thr194dup R2: p.Val193_Thr194dup R3: p.Val193_Thr194dup	R1: p.Val193_Thr194dup R2: p.Val193_Thr194dup R3: p.Val193_Thr194dup
4,39,516	*tuf1*	Synonymous	C	T	Elongation factor Tu 1	x	x	x	R3: p. His320His
5,18,130	*oadB*	Synonymous	T	C	Oxaloacetate decarboxylase beta chain	x	x	x	R3: p. Leu85Leu
14,77,186	*[ackA]–[hxpA]*	Deletion	Δ2,207βπ	x	Acetate kinase-Hexitol phosphatase	x	x	R1: Δ2,207 bp R2: Δ2,207 bp	R1: Δ2,207 bp R2: Δ2,207 bp R3: Δ2,207 bp

All the evolutionary lineages were mapped to the parent genome of *Salmonella enterica* serovar Enteritidis BAA-1045. R1, R2, and R3 refer to the three replicates in each evolutionary lineage. “X” refers to the absence of the specific mutation in that evolutionary lineage.

**FIGURE 6:**
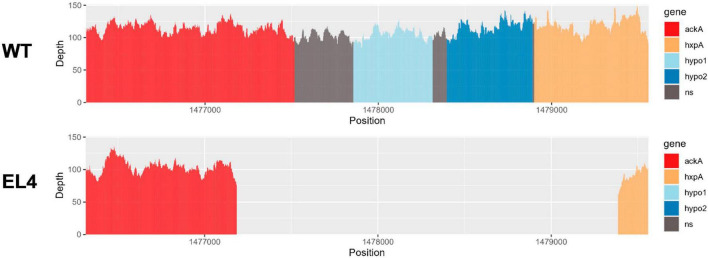
Read-depth analysis of the deletion observed between the genes *ackA* and *hxp2* in EL4. The read depth of EL4 has been compared to the wild-type (WT) strain of *S. enterica* serovar Enteritidis BAA-1045. Genes *ackA* and *hxpA* encode for acetate kinase and hexitol phosphatase in *S*. Enteritidis, respectively, and hypo1 and hypo2 stand for hypothetical protein 1 and hypothetical protein 2, respectively.

**FIGURE 7:**
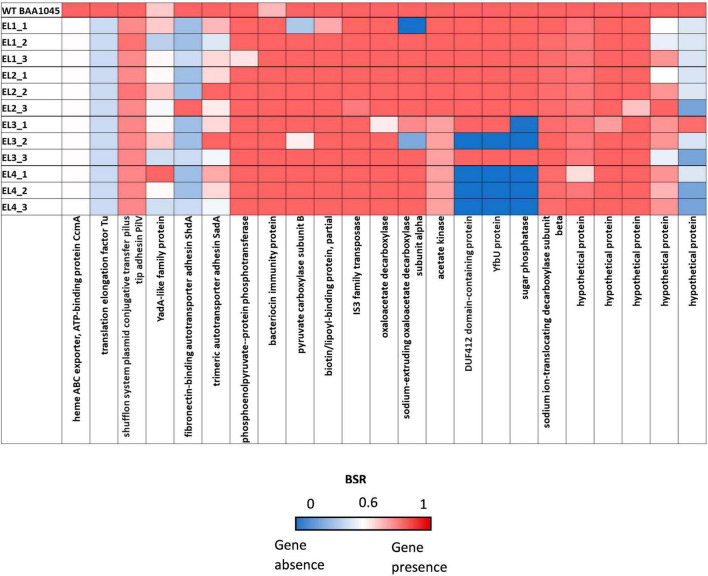
Large-Scale Blast Score Ratio (LS-BSR) heatmap of the three replicates of the evolutionary lineages EL1-EL4 after 70 ALE days.

We used the Large-Scale Blast Ratio (LS-BSR) pipeline (Sahl et al., 2014) to compare the genetic changes in the acid-adapted evolutionary lineages after 70 days. This helped us investigate the potential gain or loss of entire coding sequences in the evolved lineages (Figure 7). Additionally, a 2,207 bp deletion was observed in replicate 1 and replicate 2 of EL3 and all three replicates of EL4 (Figure 6). Read-depth analysis was used to map the deletion of genes between *ackA* and *hxpA* in EL4 and has been compared to WT *S*. Enteritidis as shown in Figure 6. This deleted region in the evolved lineage is relative to the ancestral genome as the genomic region is present in the ancestral genome, but no reads map to that region in the evolved lines, although they do map to the flanking regions.

The BLASTX and InterPro analysis suggests that there were two hypothetical proteins within the deleted range that are homologous to genes *yfbV* and *yfbU*. The read-depth analysis shows that a fraction of *ackA* and *hxpA* has been deleted, while the two hypothetical proteins *yfbV* and *yfbU* have been deleted entirely in EL4 as compared to the WT. The gene yfbV is a cytoplasmic protein that is predicted to be involved in chromosome segregation in *E. coli* (Miyakoshi et al., 2019), and the *yfbU* gene is known to play a role in DNA damage response in *E. coli* (Amitai et al., 2009). Studying the boundaries of the deleted region in EL4 indicates that by examining the extent of the reduction in read depth, it is also possible to estimate the size or length of the deleted region. This information is crucial for understanding the impact of the deletion on the affected genes. In our study, since only a fraction of *ackA* and *hxpA* is deleted, it is possible that a truncated version of the proteins is being expressed.

## 4. Discussion

This study describes the adaptation and evolution of *Salmonella* Enteritidis to different concentrations of acetic acid in TSB using adaptive laboratory evolution (ALE). The increase in MIC values of EL2, EL3, and EL4 over time suggests that *S.* Enteritidis can adapt to sub-inhibitory concentrations of acetic acid during prolonged acid exposure.

The oCelloScope is a valuable tool for studying the effects of antimicrobial compounds on bacterial growth kinetics and can be used to investigate the adaptation of bacteria to various stress conditions (Fredborg et al., 2013, 2015; McLaughlin et al., 2017). We used it to study the effects of acetic acid stress on the exponential hourly growth rate of the evolved lineages and compared them to the growth rate of WT *S.* Enteritidis. Our results indicate that the growth rates of the evolved lineages in the presence of acetic acid were dependent on the concentration of acid used during the evolutionary process (Figure 3, Table 1 and Supplementary Figure 2). Since EL1 was adapted without any acid stress throughout the ALE study, there were no significant differences in the exponential growth rate of its three replicates between ALE days 2 and 70 after exposure to 26 mM acetic acid in the oCelloScope. The exponential growth rates of EL1 recovered from days 2 and 70 in the presence and absence of 26 mM acetic acid were 0.2 (h^–1^) and 0.4 (h^–1^), respectively. These values were similar to the exponential growth rate of WT *S.* Enteritidis in the presence and absence of 26 mM acetic acid as monitored using the oCelloScope. The growth rates of EL2–EL4 on ALE day 70 were significantly higher than the values 2 days after the initiation of the corresponding evolutionary lineages (*p* < 0.01). Other researchers have demonstrated that adaptation in stress environments caused an increase in the growth rate of *E. coli* during ALE studies (Fong et al., 2005; Cheng et al., 2014; LaCroix et al., 2015).

Next, we aimed to investigate whether long-term exposure to acetic acid in the evolutionary lineages led to cross-resistance against human antibiotics (Figure 4). The different classes of antibiotics used in this study against *S. enterica* have been tested in earlier studies (Geornaras and von Holy, 2001; Jones et al., 2002; Muhammad et al., 2010; Chang et al., 2021). Studies in *E. coli* have found that resistance against antibiotics changes over the course of ALE studies (Maeda et al., 2020; Marciano et al., 2022). *E. coli* strains sensitive to carbapenem were found to develop resistance against carbapenems by the 15th generation in another ALE study (Geng et al., 2022). Ciprofloxacin has historically been used as the first line of defense against *S. enterica* infections, especially against many multidrug-resistant (MDR) strains (Askoura and Hegazy, 2020). Carbapenems (meropenem) have also been used successfully for the treatment of *S. enterica* infections, especially the ones that are resistant to cefotaxime and ciprofloxacin (Jean et al., 2005). Using antibiotics with distinct mechanisms of action provides us with a more comprehensive understanding of the evolution of resistance in evolved populations.

We did not observe significant changes in the MIC of the antibiotics tested against EL1 between days 2 and 70, likely due to the lack of any selective pressure on EL1 in the ALE study. In contrast, significant increases in the MIC values of ciprofloxacin, gentamycin, and meropenem against EL 4 (*p* < 0.05) (Figure 4 and Supplementary Table 1) were observed after day 70. This highlights that exposure to acid stress provides cross-adaptation against antibiotic stress in *S. enterica* acid-evolved populations. This result is in line with previous studies demonstrating that exposure to low concentrations of stress over time in *S. enterica* leads to increased MIC against antibiotics (Gullberg et al., 2011; Wistrand-Yuen et al., 2018).

Even though the MIC against the various antibiotics, especially, ciprofloxacin, gentamycin, and meropenem, was found to significantly increase after 70 ALE days, their resultant MIC values still fell within the susceptible range as defined by established clinical breakpoints (FDA, 2023). Despite this, there remains a pertinent cause for concern regarding the increase in MIC values as this suggests that the ELs exposed to acid stress displayed a reduced susceptibility to the antibiotics over time. The rise in MIC values, albeit within the susceptible range, potentially signifies the presence of underlying mechanisms that contribute to reduced antibiotic effectiveness. Such alterations in MIC values hint at adaptive responses in *S. enterica* under acid stress, which could subsequently impact the overall efficacy of antibiotic treatment strategies. This finding also raises concerns about the potential for acidic conditions, such as those encountered during food processing, which may inadvertently contribute to the development of antibiotic-resistant bacterial populations.

The observed differences in growth rate and increase in MIC of acetic acid and antibiotics over time suggest the presence of genetic changes enabling the ELs to adapt to the selective pressure of acid. Long-term exposures to acid stress have been reported to be associated with mutations at the genomic level in *E. coli* (Du et al., 2020; Zeng et al., 2021), which can alter the bacterial cell structure, physiology, and metabolism leading to multidrug resistance (MDR). However, when the ELs from day 70 were desiccated on the surface of stainless-steel coupons for 18 h and subsequently exposed to 100 mM acetic acid using previously established methods (Ghoshal et al., 2022a), no significant differences between the log reduction of the ELs and WT *S*. Enteritidis were observed (Supplementary Figure 3). This could be due to the fact that bacterial adaptation to a specific stressor often involves the optimization of cellular mechanisms and regulatory pathways to withstand the stress within a limited concentration range (Mongold et al., 1999). In the case of acetic acid, the acid-adapted bacteria may have undergone genotypic and phenotypic changes such as alterations in membrane permeability, efflux pump activity, intracellular pH regulation, and activation of stress response systems that enable them to survive and proliferate in the presence of lower concentrations of acetic acid. However, when exposed to significantly higher concentrations, the stress exceeds the threshold of their adaptive response, leading to a loss of viability and growth inhibition.

Mutations in the outer membrane lipoproteins and membrane transporters are known to alter the binding properties of bacterial membranes which increase resistance against various antibiotics such as vancomycin and meropenem (Langsrud et al., 2004; Maldonado et al., 2016). We observed mutations in the outer membrane lipoprotein *ybaY* as well as mutations in the outer membrane transporter *fhuA* in the acid-adapted *S*. Enteritidis lineages, and it is possible that these mutations rendered the outer membrane less susceptible to antibiotics. Additionally, periplasmic binding proteins help gram-negative pathogens sense their environment, regulate the uptake of small molecules, and even provide antibiotic resistance in *S. enterica* (Blair et al., 2009). Mutations observed in the thiamine-binding periplasmic protein in EL3 and EL4 as well as in the metal-binding protein *zinT* in EL2 could have possibly played a role in providing antibiotic cross-resistance after acid adaptation.

In *E. coli*, exposure to antimicrobial compounds such as benzalkonium chloride (BAC) has been reported to induce cross-resistance against other antimicrobial compounds and antibiotics (Chapman, 2003; Langsrud et al., 2004). The acquisition of antibiotic resistance in these studies was mainly attributed to the differential expression of bacterial efflux pumps and several outer membrane proteins. Changes in the expression of efflux pumps promote resistance against several classes of antibiotics (Jibril et al., 2021; Dawan et al., 2022). A variety of *Salmonella* strains also differentially express enzymes that can modify and inactivate antibiotics such as streptomycin (Mengistu et al., 2020). Upregulation in the expression of several transcription factors, which are global regulators of stress, is also known to trigger multidrug resistance in *E. coli* (Duval and Lister, 2013). Future RNA sequencing studies on the acid-adapted lineages of *S*. Enteritidis will help to determine the influence of efflux pumps, membrane proteins, and transcription factors in providing cross-resistance against antibiotics. The two-component signal transduction system *PhoP/Q* is reported to provide general cellular defense against stressful conditions and antibiotic resistance in *E. coli* and *S.* Enteritidis (Lázár et al., 2014; Hu et al., 2023). A hypothesis is that the mutations observed in *phoP* and *phoQ* observed in our study played an important role in triggering cross-adaptation against antibiotics in the acid-evolved *S. enterica* lineages.

Additionally, the phenomenon of persistence is triggered in bacteria after exposure to various stressful environments which renders them resistant to future stressors such as exposure to antibiotics (Pacios et al., 2020). Persistence is known to provide *E. coli* with tolerance and resistance against several antibiotics (Li and Zhang, 2007), and short-term exposure to acid stress is reported to trigger persistence in *S. enterica* (Leyer and Johnson, 1992). We hypothesize that reduced susceptibility to the antibiotics could be in part due to the presence of persister cells in the subpopulation of the acid-evolved *S. enterica* lineages. It is also possible that cross-resistance to antibiotics in *S.* Enteritidis acid-evolved lineages is due to a combination of the factors described above.

Genomic analysis of the acetic acid-evolved ELs revealed base substitutions in 15 different genes (Table 2), including missense mutations in multiple ELs in *phoQ* and *phoP*. The PhoQ protein is known to control the transcription of a large class of genes (Prost et al., 2007), and the PhoP/Q system is triggered under conditions of low pH and in the presence of antimicrobial and cationic peptides in *S. enterica* (Bearson et al., 1998; Prost et al., 2007; Yuan et al., 2017). Activation of this system is known to regulate *S. enterica* virulence and pathogenesis (Prost et al., 2007). In addition, the PhoP/Q protein system has been reported to be involved in stress resistance and antibiotic resistance and in the regulation of envelope composition in *S. enterica* (Yuan et al., 2017; Shprung et al., 2021; Dawan and Ahn, 2022).

The presence of mutations in the *phoP/Q* genes in all three replicates of EL2, EL3, and EL4 and its absence in EL1 indicate that these mutations are a result of the adaptation to acetic acid stress. One of the mutations observed in *phoQ* in EL2 was in the HAMP domain of the protein (Figure 5), which is thought to play an important role in transmitting signal to the catalytic domain of the protein (Lemmin et al., 2013). Base substitutions in the protein PhoQ Asn255Ileu have been reported where an amino acid with a neutral polar R group was replaced by a neutral non-polar R group under stress conditions (Matamouros et al., 2015) which is similar to our case (Thr271Pro). Researchers found that the Asn255Ileu substitution significantly increased the activity of the PhoQ protein (over 10-fold increase in activity) and that the mutant protein was found to spend more time in the activation state than the wild-type protein (Matamouros et al., 2015). It is possible that the mutation in our system also led to a similar increase in the activity of the PhoP protein. We also observed base substitutions in the sensor histidine kinase domain of *phoQ* in EL3 and EL4, which serves as the catalytic domain and regulates/activates the phosphoryl-state of the protein (Lemmin et al., 2013). Additionally, we observed a mutation in EL2 in the OmpR/PhoB-type DNA-binding domain of *phoP* (Figure 5). In this case, arginine, which is a basic amino acid, was replaced by glutamine, which is polar in nature, and at low pH, such amino acids acquire an overall positive charge. This positively charged amino acid in DNA-binding domains of proteins aids in more effective binding with negatively charged DNA (Cherstvy, 2009), and such modifications in response regulators are often associated with differential changes in gene expression of downstream genes (Rhee et al., 2008). It is possible that the mutations in *phoP/Q* among the ELs contributed to the reduced susceptibility against the antibiotics causing a differential expression of stress and acid adaptation genes. Mutations in the *phoPQ* system are associated with increased resistance against antibiotics in other gram-negative pathogens such as *Pseudomonas aeruginosa* (Miller et al., 2011). In our study, we have chosen to prioritize the investigation of missense mutations within the *phoP/Q* genes as a primary focus, while avoiding gene deletion studies. This strategic decision is grounded in several considerations. First, the existing body of literature provides substantial evidence suggesting that missense mutations in *phoP/Q* genes play a pivotal role in bacterial adaptations to stress conditions, including acid stress (Prost et al., 2007; Matamouros et al., 2015; Pitt et al., 2018). Moreover, planned RNA sequencing studies in future will offer valuable insights into the dynamic changes in the expression of *phoP/Q* genes in response to acid stress. These findings will complement our observations of missense mutations and help establish a multifaceted understanding of the molecular mechanisms underlying the adaptation process. By focusing on missense mutations in conjunction with transcriptomic analysis, we aim to provide a comprehensive perspective on the significance of *phoP/Q* genes in stress adaptation without resorting to gene deletions. This will help minimize potential disruptions to the overall cellular machinery and preserve the ecological relevance of our study.

In EL2, we found a mutation in the *fhuA* gene which encodes for the ferrichrome outer membrane transporter. This mutation was present in all three replicates of EL2, suggesting that it was not random and probably associated with the adaptive evolution in acetic acid. The gene *fhuA* is involved in the transport of antibiotics such as albomycin and rifamycin, and bacteriophages and toxins such as colicin M inside *E. coli* cells (Braun et al., 2002). Mutations in this gene have been reported in *E. coli* and *S. enterica* under stress conditions, which enable the bacteria to escape from future attacks of antibiotics while maintaining its siderophore uptake activity that is essential for the survival of the bacteria (Wang et al., 2018). Additionally, two of the three replicates of EL2 showed mutations in the *rpoB* gene, which is usually targeted by antibiotics such as rifampicin in *E. coli* and *S. enterica* (Björkman et al., 1998). Several studies have shown that mutations in the *rpoB* gene lead to resistance against rifampicin and other similar antibiotics in *E. coli* and *S. enterica* (Björkman et al., 1998; Garibyan et al., 2003; Rodríguez-Verdugo et al., 2013). Mutations in *rpoB* are also known to be triggered during stress conditions in antibiotic-free environments (Rodríguez-Verdugo et al., 2013). This is similar to our study, where mutations in *rpoB* gene were triggered during adaptation to acid stress in the absence of antibiotics. Another gene that was mutated in all three replicates of EL3 and EL4 is *rfbE*, which is involved in the synthesis of the O-antigen in *E. coli* and the *rbf* gene cluster is known to be involved in the synthesis of the O-antigen of cell wall LPS in *S. enterica* and other gram-negative Enterobacteriaceae (Iguchi et al., 2011; Cota et al., 2015). The O-antigen is involved in the pathogenesis and virulence of *S. enterica* (Murray et al., 2003). Mutations in the O-antigen in the cell wall LPS are likely to affect the conformation and function of various surface proteins. The composition of the O-antigen affects bacterial surface charge, which is known to alter membrane permeability under changing pH conditions (Lerouge and Vanderleyden, 2002). We measured the surface charge of the ELs after 70 ALE days in acid stress and compared them to the surface charge of WT *S*. Enteritidis to study whether acid adaptation caused any changes in the surface charge. No significant differences in the surface charge of the ELs were observed as compared to the WT *S*. Enteritidis after 70 ALE days (*p* > 0.05) (Supplementary Table 2). Furthermore, the gene *thiB* that encodes for the thiamine-binding periplasmic protein was found to be mutated in the three replicates of EL3 and EL4. The presence of environmental stresses has been reported to cause mutations in genes involved in thiamine biosynthesis and transport in yeast cells (Kartal et al., 2018).

Finally, the deletion of 2207 bp of the genome in two replicates of EL3 and all three replicates of EL4 was observed. There were two major genes in this region, namely, *ackA*, which encodes for acetate kinase, and *hxpA*, which encodes for hexitol phosphatase. The gene *ackA* encodes for acetate kinase that is involved in the production of acetate from acetyl phosphate (Kumari et al., 2000). Deletion of this gene could be attributed to the presence of large amounts of acetate available during acetic acid stress, and hence, the need for this enzyme was significantly reduced in the ELs. Even though the *ackA* gene is also involved in the conversion of acetate to acetyl phosphate, which is a precursor to acetyl-CoA, there is an alternative pathway for the synthesis of acetyl-CoA from acetate (Kumari et al., 1995). This pathway is catalyzed by the enzyme acetyl-CoA synthase, and it is commonly used by cells during stressful conditions such as nutrient limitation, low oxygen pressure (De Mets et al., 2019), and, possibly, acid stress.

Since EL2 is the primary ancestor of the evolutionary lineages EL3 and EL4, we studied the genomes of EL2–EL4 to look for any common mutations that may have occurred before ALE day 20 and subsequently passed on to EL3 and EL4. We did not observe any common mutations in EL2–EL4 suggesting that most of the mutations observed in EL2 happened after ALE day 20 when EL3 diverged from EL2. Even though replicate 2 of EL2 and all three replicates of EL3 and EL4 had mutations in the *phoQ* gene, the positions of this mutation in EL3 and EL4 were different from EL2, indicating these occurred after ALE day 20. Missense mutations in *rfbE*, *phoQ*, and *thiB* were identical between EL3 and EL4, which suggests that these mutations occurred between ALE day 20 (after the split of EL3 from EL2) and ALE day 30 (before the initiation of EL4 from EL3). SNPs in certain genes such as *tuf1* and *oadB* were observed in EL4 alone, indicating these mutations occurred after ALE day 30 when EL4 was split from EL3. EL3 and EL4 demonstrated a 2207 base pair mutation between genes *ackA* and *hxpA*. In EL3 and EL4, this mutation was observed in replicates 1 and 2, which possibly indicates that they were carried from EL3 to EL4.

Although our study is one of the first of its kind to study acid adaptation in *S. enterica*, prior studies have revealed the effects of acid adaptation in *E. coli*, which is a related gram-negative pathogen. Harden et al. (2015) documented that *E. coli* K12 lineages exposed to acid conditions exhibit enhanced growth rates and improved fitness. The same phenomenon was replicated in our investigation, where we observed an increase in growth rates among acid-evolved *S. enterica* lineages when subjected to acidic conditions. Other researchers have also independently corroborated these findings by demonstrating increased growth rates and enhanced survival in acid-evolved *E. coli* lineages exposed to acidic environments (He et al., 2017; Du et al., 2020). These researchers also reported that the acid-evolved *E. coli* lineages had mutations in the RNA polymerase subunits. Our study supports these findings as we also observed mutations in the RNA polymerase subunit beta in EL2. These findings suggest that RNA polymerases play an important role in acid stress adaptation in many gram-negative pathogens. Mutations in the RNA polymerase can lead to changes in gene expression, which could affect the production of acid-resistant proteins, the transport of protons out of the cell, or other cellular processes that are important for acid survival.

The results of this study highlight that adaptation to acetic acid stress is associated with genotypic and phenotypic changes in *S. enterica*. Similar genetic and phenotypic changes in *Salmonella* are expected if other organic acids with similar chain lengths and mechanisms of action against *S. enterica* were used during the adaptive process. The increased MIC of the ELs against both acetic acid and human antibiotics raises concerns regarding the development of cross-resistance and the potential emergence of multidrug-resistant *S. enterica.* Although the specific mechanisms associated with the cross-resistance remain to be elucidated, it is plausible that the mutations identified in the *phoPQ* system and other stress-related genes along with the gene deletions contribute to a broader adaptive response, leading to increased resistance against multiple stressors, including antibiotics.

## 5. Conclusion

Understanding the genetic mechanisms associated with acid stress adaptation can lead to targeted interventions and therapies to combat antimicrobial resistance. The uniqueness of our study lies in the fact that it is the first of its kind (to the best of our knowledge) to apply adaptive laboratory evolution for studying the adaptation of *S. enterica* under acid stress. The findings of this study have implications for food safety and demonstrate the utility of the ALE approach in studying the evolution and adaptation of foodborne pathogens to environmental stresses, particularly acid stress. Organic acids, especially acetic acid, are often used as additives for flavoring and preserving different foods and for cleaning and sanitization (Mani-López et al., 2012; Coban, 2020). It is possible that *S. enterica*, present as a contaminant in the food industry, is exposed to sub-lethal levels of acetic acid. The results of this study highlight that under such conditions, *S. enterica* will be able to adapt and evolve in acid stress. This might lead to enhanced persistence and survival of the bacteria, leading to a higher risk of contamination of food products during processing. The increase in MIC against human antibiotics in the acid-adapted *S. enterica* strains can also pose a significant public health risk. The results of this ALE study emphasize the need for continuous evaluation and optimization of sanitization protocols to ensure their efficacy against emerging antibiotic-resistant pathogens. Finally, this study underscores the need for comprehensive approaches to combat antimicrobial resistance, including the prudent use of acid-based antimicrobials and the development of alternative antimicrobial strategies.

## Data availability statement

The datasets presented in this study can be found in online repositories. The names of the repository/repositories and accession number(s) can be found in the article/Supplementary material.

## Author contributions

MG: Conceptualization, Data curation, Investigation, Methodology, Writing – original draft, Writing – review and editing. TB: Formal analysis, Methodology, Writing – original draft. JG: Conceptualization, Software, Supervision, Visualization, Writing – review and editing. LM: Conceptualization, Funding acquisition, Methodology, Project administration, Resources, Supervision, Writing – review and editing.
