# Supplementary material for: Adaptive laboratory evolution of Salmonella enterica in acid stress
Source: Front Microbiol. 2023 Nov 16;14:1285421. doi: 10.3389/fmicb.2023.1285421 (PMC10687551; doi:10.3389/fmicb.2023.1285421)
Supplement: Supplementary file 1 [file Data_Sheet_1.pdf]

Supplementary Tables

Supplementary Table 1. Antibiotic sensitivity of adapted evolutionary lines.

Evolutionary lines	Ciprofloxacin		Gentamycin		Meropenem		Streptomycin		Vancomycin	
	**D2**	**D70**	**D2**	**D70**	**D2**	**D70**	**D2**	**D70**	**D2**	**D70**
EL1a	0.016	0.016	0.38	0.38	0.047	0.047	2	2	0.5	0.5
EL1b	0.016	0.016	0.38	0.38	0.047	0.047	2	2	0.5	0.5
EL1c	0.016	0.016	0.38	0.38	0.047	0.047	2	2	0.5	0.5
	**D2**	**D70**	**D2**	**D70**	**D2**	**D70**	**D2**	**D70**	**D2**	**D70**
EL2a	0.016	0.016	0.38	0.38	0.047	0.047	2	2	0.5	0.75
EL2b	0.016	0.016	0.38	0.38	0.047	0.047	2	2	0.5	0.50
EL2c	0.016	0.016	0.38	0.38	0.047	0.047	2	2	0.5	0.50
	**D22**	**D70*** **	**D22**	**D70**	**D22**	**D70**	**D22**	**D70**	**D22**	**D70**
EL3a	0.023	0.032	0.5	1.0	0.047	0.064	2	4	0.5	0.50
EL3b	0.023	0.032	0.5	0.75	0.047	0.047	2	4	0.5	0.75
EL3c	0.023	0.032	0.5	0.75	0.047	0.064	2	6	0.5	0.50
	**D32**	**D70***	**D32**	**D70***	**D32**	**D70*****	**D32**	**D70**	**D32**	**D70**
EL4a	0.032	0.047	0.5	0.75	0.047	0.094	3	6	0.5	0.75
EL4b	0.032	0.047	0.5	1.0	0.047	0.094	3	6	0.5	1.0
EL4c	0.023	0.047	0.5	1.0	0.047	0.094	3	6	0.5	0.75

Footnote: MIC of the antibiotics against the evolutionary lineages in µg/ml. EL 1(a-c) was adapted in 0mM acetic acid, EL2 (a-c) was adapted in 26mM acetic acid, EL3 (a-c) was adapted in 28mM acetic acid and EL4 (a-c) was adapted in 30mM acetic acid.

MIC of the antibiotics have been quantified on ALE Day 2 and Day 70 for EL1 and EL2, between ALE day 22 and day 70 for EL3, and ALE day 32 and Day 70 for EL4.

For EL3 (a-c) and EL4 (a-c) Day 22 and Day 32 were 2 days after the start of the evolutionary lines respectively. MIC of ciprofloxacin in EL3 on day 70 was significantly higher than that on day 22. MIC of meropenem, ciprofloxacin and gentamycin for EL4 on day 70 were significantly higher than that on day 32. Asterisks (*) in the table indicates significant differences in growth rates in comparison to controls (* $p<0.05$ and *** $p<0.001$).

Supplementary Table 2: Bacterial cell surface charge (mV), zeta potential

**Evolutionary lines**	**Bacterial cell surface (mV)**	
WT	-13.4	
	**Day 2**	**Day 70**
EL1a	11.3	13.1
EL1b	12.5	10.8
EL1c	9.7	12.4
	**Day 2**	**Day 70**
EL2a	10.4	12.6
EL2b	8.6	10.5
EL2c	9.4	12.9
	**Day 22**	**Day 70**
EL3a	9.4	12.5
EL3b	11.4	13.7
EL3c	10.6	12.3
	**Day 32**	**Day 70**
EL4a	9.2	13.2
EL4b	11.9	10.8
EL4c	12.4	11.1

Footnote: The cell surface charge of WT S. Enteritidis and the EL quantified using the Zeta Master
