# Supplementary material for: Adaptive laboratory evolution of Salmonella enterica in acid stress
Source: Front Microbiol. 2023 Nov 16;14:1285421. doi: 10.3389/fmicb.2023.1285421 (PMC10687551; doi:10.3389/fmicb.2023.1285421)
Supplement: Supplementary file 2 [file Data_Sheet_2.pdf]

Supplementary Figures

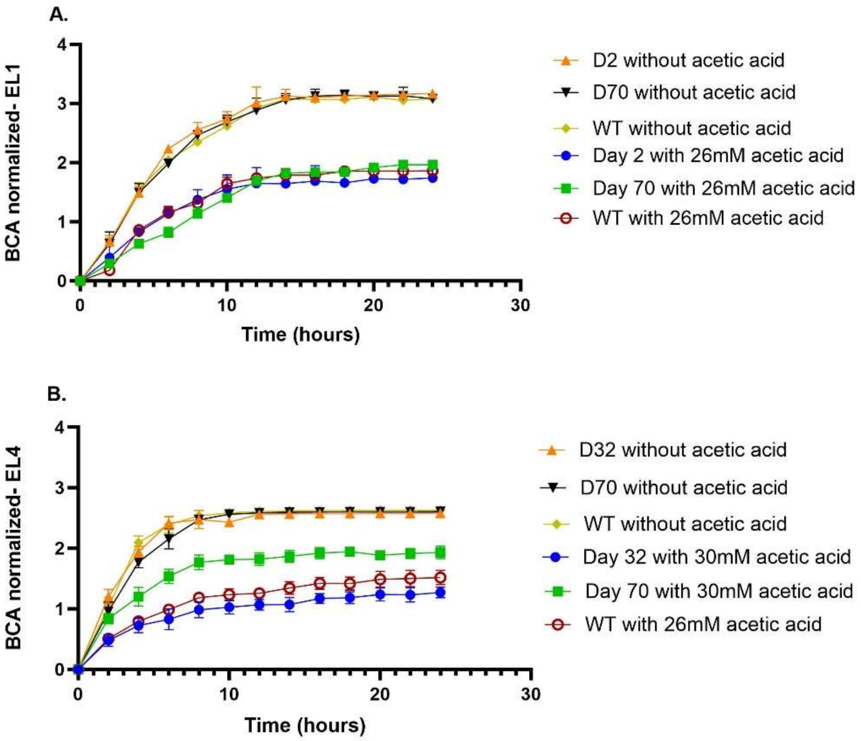

Supplementary 1: Growth curve of EL1 (A) and EL4 (B) in acetic acid generated using the normalized values of background corrected absorption (BCA) algorithm of the oCelloScope.

EL1 was adapted without acid stress for 70 days while EL4 was adapted in 30mM acetic acid starting from day 30 to day 70 of the ALE study.

For EL1 and EL4, inoculum was taken after 2 days of daily passages (Day 2 for EL1 and Day 32 for EL3) and after day 70 of the ALE study. The evolutionary lineages were grown in the oCelloScope in sub-MIC of acetic acid for each. Hence, EL1 was grown in 26 mM acetic acid while EL4 was grown in 30mM acetic acid. The growth curves of EL1 and EL4 have been compared to WT S. Enteritidis which was also grown in 26mM acetic acid since its MIC of acetic acid was also 27mM. Growth rates have been calculated using the BCA values from the exponential phase of the growth curves.

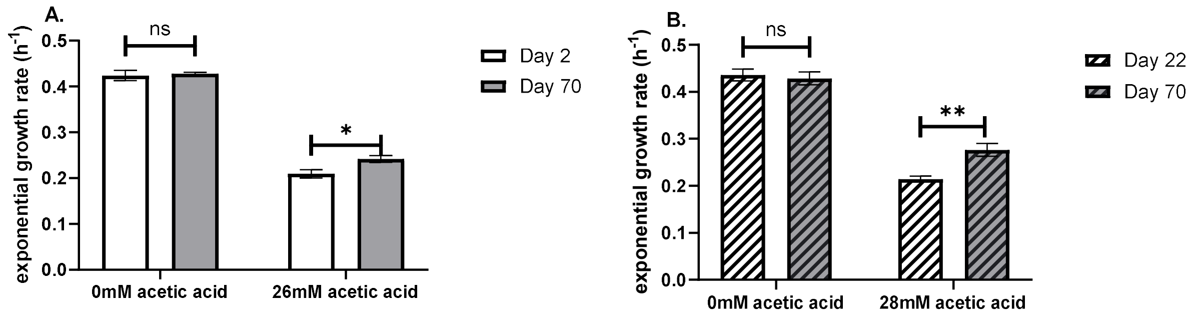

Supplementary 2: Change in growth rate of EL2 (A) and EL3 (B) in the presence and absence of acetic acid 2 days after the initiation of the EL (Day 2 for EL2 and Day 22 for EL3) and after day 70 of the ALE study.

The evolutionary lineages were grown in the oCelloScope in sub-MIC of acetic acid for each lineage. Hence, EL2 was grown in 26 mM acetic acid while EL3 was grown in 28mM acetic acid. p-value of 0.05 was considered to be statistically significant using two-tailed t-test. (**= p< 0.01, ns= non-significant= p >0.05)

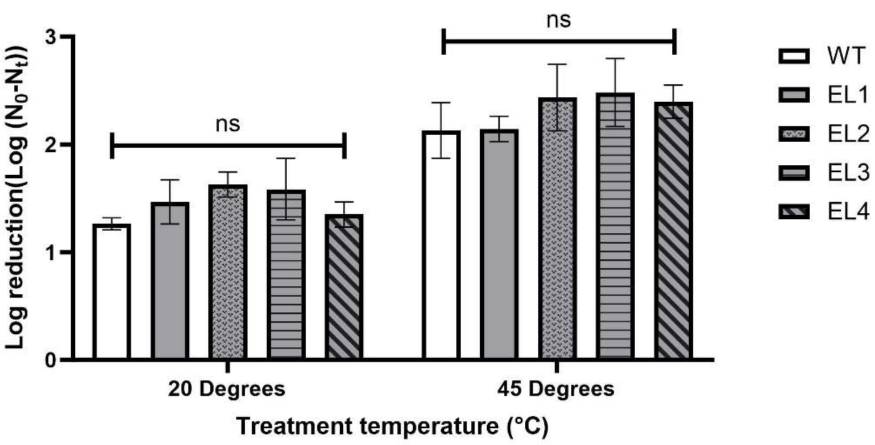

Supplementary 3: Log reduction in the presence of acetic acid in oil (100mM) in WT S. Enteritidis and EL1-EL4 on day 70.

p-value of 0.05 was considered to be statistically significant using two-tailed t-test. (ns= non-significant= p >0.05).
